# Effects of playing position, pitch location, opposition ability and team ability on the technical performance of elite soccer players in different score line states

**DOI:** 10.1371/journal.pone.0211707

**Published:** 2019-02-05

**Authors:** Athalie J. Redwood-Brown, Peter G. O’Donoghue, Alan M. Nevill, Chris Saward, Caroline Sunderland

**Affiliations:** 1 Sport, Health and Performance Enhancement Research Centre, Department of Sports Science, Nottingham Trent University, Nottingham, United Kingdom; 2 Cardiff Metropolitan University, Cardiff, United Kingdom; 3 Faculty of Education, Health and Wellbeing, Wolverhampton University, Wolverhampton, United Kingdom; Queen Mary University of London, UNITED KINGDOM

## Abstract

The purpose of this study was to investigate the effects of playing position, pitch location, team ability and opposition ability on technical performance variables (pass, cross, corner, free kick accuracy) of English Premier League Soccer players in difference score line states. A validated automatic tracking system (Venatrack) was used to code player actions in real time for passing accuracy, cross accuracy, corner accuracy and free kick accuracy. In total 376 of the 380 games played during the 2011–12 English premier League season were recorded, resulting in activity profiles of 570 players and over 35’000 rows of data. These data were analysed using multi-level modelling. Multi-level regression revealed a “u” shaped association between passing accuracy and goal difference (GD) with greater accuracy occurring at extremes of GD e.g., when the score was either positive or negative. The same pattern was seen for corner accuracy away from home e.g., corner accuracy was lowest when the score was close with the lowest accuracy at extremes of GD. Although free kicks were not associated with GD, team ability, playing position and pitch location were found to predict accuracy. No temporal variables were found to predict cross accuracy. A number of score line effects were present across the temporal factors which should be considered by coaches and managers when preparing and selecting teams in order to maximise performance. The current study highlighted the need for more sensitive score line definitions in which to consider score line effects.

## Introduction

There has been much speculation about the influence of score line (i.e. scoring and conceding goals and/or whether a team is winning, drawing or losing) on player performance [1,2,3,4,5]. Such speculation has motivated academic researchers [1,2,3,4,5] to ascertain the influence of score line on different aspects of sports performance. Score line is generally defined as winning, losing or drawing state, however more recently smaller data sets (e.g. World Cup Tournaments) have included specific score lines or goal differences (GD) (e.g., 1:0, 1:1, 2:0 etc.) in an attempt to understand how the size of the lead or deficit affects player performance [6,7].

In soccer, the score line has been found to influence both technical (e.g. passing accuracy, successful possessions and successful throw-ins) [1,6,7,8,9,10] and tactical performance (e.g. passing patterns, possession length and number of shots on target) [5,7]. Although previous research has generally found variables such as passing accuracy and possession to vary depending on whether a team is winning, losing or drawing [5] many studies come under criticism due to the methods used to collect data. One of the main criticisms has been the subjective nature of many of the methods used to investigate such performance factors for example, where human operators have coded matches with little or no confirmation of the accuracy of the data [11]. This has led to inaccuracies and errors when recording data, questioning the reliability of findings from these studies [11,12]. More recently, researchers [7,13] have used established industry definitions to try to give wider application and greater validity to findings. However, this does not eradicate the problems associated with human error when identifying events, especially in real time. This subjective nature of human event identification is also time consuming, therefore limiting both the number of games that can be included and the number of players observed. Advancements in technology (such as computerised tracking systems) have enabled researchers [6, 13,14,15,16,17] to analyse match performance from a physical perspective (e.g. investigating players activity profiles) however the use of such systems when investigating technical or tactical performance factors is scarce. Previous technological barriers in data collection methods have also limited the ability to generalise findings for both physical and technical performance variables. For example, categorising players by position (defenders, midfielders, attackers) in relation to score line effects has only been considered using small data sets or single clubs [16] using overall match status (wining, drawing or losing) rather than by how much a team is winning or losing by (i.e., the goal difference). Using an automated tracking system dramatically reduces the time taken to categorise technical player actions as well as ensuring more accurate player identification. Such automated methods such as that validated by Redwood-Brown et al. [17] would enable a greater volume of players to be observed as well as further investigating how players in different playing positions react under different score line states across multiple games and when playing teams of different standards. The use of such methods also allows for pitch locations to be calculated using calibrated pitch positions, further adding to the reliability of the data observed. This is especially important, as technical factors (e.g. passes, free kicks etc.) have been found to vary in different pitch locations as a function of the score line [7], and across different playing position [18] and team abilities [9,19,20].

A secondary issue of previous research [3,13,21,22,23,24] has been the failure to consider normal performance, e.g. how teams perform when no goals are scored and teams are of similar ability. For example, a team may score in the first 5 minutes of the game and spend the remaining time defending their lead and thus passing successful rate maybe a function of the team’s strategy rather than an accurate representation of performance. Technical and tactical factors may also vary as a function of the opposition’s ability especially as previous research has found that teams decrease their passing accuracy when winning and increase passing accuracy after conceding [2,6,7]. Using a more sensitive definition of both score line and opposition ability may help managers and coaches to better understand the effect that the score line may have on technical performance factors when playing different standards of opposition. Using automated tracking to time stamp specific performance variables such as passes, crosses, corners, free kicks, etc. will also help to establish normative data for games where teams spend long periods of time in different score lines states.

Redwood-Brown et al. [25] recently highlighted the impact of psychological factors on player performance with regards to scoring and conceding. Their findings along with previous research [26] suggest that if the outcome of a match becomes obvious during the second half (e.g., the opposition are of a higher standard), player motivation might be reduced, potentially leading to a reduction in effort and thus a reduction in the amount of time chasing the ball or attempting to regain possession [3,26]. Although reduction in effort (normally defined as fatigue) has been considered in recent studies with regards to physical variables such as distances covered [18,21,27,28], the sample size and subjective nature of the data collection methods has limited the application of the findings and there is still a lack of consideration with regards to ‘effort’ when observing technical or tactical variables.

A third issue has been the lack of consideration to the match location (e.g., whether teams are playing at home or away) with regards to score line, especially given the volume of research that has highlighted this as a factor relating to success in soccer [5,9,26,29]. For example, Lago and Martin [9] found home teams had greater possession than their opposition, a pattern found by numerous studies across a wide range of playing abilities [1,5,8,30,31,32]. Tucker et al. [31] and Taylor et al. [32] also suggested that home teams tended to perform a higher number of attacking actions (goal scored, shots on goal, passes, crosses etc.) which is not surprising given research investigating match location effects has suggested that the home team have a number of advantages over the visiting team [31,33,34]. Home advantage has also been found to produce triggers for positive momentum (e.g. crowd effects) [35,36,37] as supporters are typically in a win frame (e.g. focused on achieving success), thus motivate teams to perform [26]. Lago-Peñas and Dellal [2] found that higher ranked teams had less variation in performance than lower ranked teams suggesting that higher ranked teams are able to maintain their performance regardless of the environment and situation (playing at home or away/losing, winning, drawing). Therefore, it is essential to account for match location in order to establish the effects of score line on soccer performance. Investigating the interaction between match location and temporal factors with regards to score line would help managers and coaches to further understanding their impact on technical performance and plan accordingly.

The aim of the present study was to investigate how players’ performances vary with a number of situational factors (playing position, opposition ability, team ability, pitch location and time scored) at different score-line states both at home and away from home. The use of an automated tracking system [17] will also allow the aggregated data of several teams to be analysed rather than a single team thus creating more normative data to improve team performance in a collective way [38].

## Methods

### Ethics statement

This study was approved by the School of Science and Technology non-invasive Ethics Committee at Nottingham Trent University.

### Data set

In total 376 of the 380 games played during the 2011–2012 English Premier League season were used in the current study which included 570 players and 35’000 rows of data. The omission of four games was due to a number of technological incidents outside of the operators’ control, which disabled the system and resulted in the tracking data becoming unusable. This resulted in 20 teams who played against each other at both their own ground and that of their opponents, with the exception of the teams affected by the excluded games. The ability of each team and their respective opponents was calculated using their final league position (ranked 1–20, i.e. 1^st^ in the league to 20^th^ in the league) at the end of the season once all games had been played. This was in line with previous research [5] which has highlighted the need for greater sensitively when using ability as a situational factor relating to team performance. For accuracy player position (striker, midfielder, defender) was determined at the start of each game using the official team sheets provided to the press association. This ensured players who may change positional role depending on the tactical strategy adopted by the team were accurately defined for each game. The pitch was split evenly into three sections (attacking third, middle third and defensive third) [7] using a theodolite and calibrated pitch dimensions (specific to each individual stadium). Consent to use the data for research purposes was provided by both Venatrack Ltd and the English Premier League.

### Data gathering

Data were recorded using the live broadcasting feed provided by the host broadcaster and Venatrack’s live eventing system. Two trained observers, event analysis A (EAA) and event analysis B (EAB) used the live eventing system to code game events alongside the automated tracking system (Venatrack. Ltd.) [17]. This enabled positional and speed parameters to be related to coded game events. Any ambiguous outcomes were flagged on the system and discussed with the team of analysts at the end of the game prior to submitting the final eventing report to ensure reliability, and validity of the data. The video capture system used 28 HD colour cameras positioned at specific locations around the respective soccer stadium. Twenty Eight HD cameras were used to ensure that the maximum positional accuracy (visual acuity) was provided to the computer algorithm. The estimated visual acuity for the current system was in the range 5 – 25mm compared to previous systems, which have been estimated at between 500mm– 1500m depending on the region of the pitch. The cameras’ position, orientation and field of vision were determined and fixed using a Theodolite (Nikon NPL 362, Japan) during installation. The cameras were positioned to give a full view of the pitch using the systems unique configuration co-ordinates (unique to each ground), which allowed each position on the pitch to be covered by at least five cameras at any one time (Venatrack Ltd, UK). Calibration of the automatic tracking system was completed by a team of technical experts who had collectively over eighteen years of experience of visual artificial intelligent (AI) technology, such as that used by the system in question. The system was also found to be valid and reliable for tracking player movement at both high speed and sprinting distances [17].

#### Performance indicators (technical and tactical)

A number of technical performance indicators were used to describe player performance. In order to ensure accuracy and reliability of these performance variables strict definitions were used for the four technical indicators (passes, crosses, corners and free kicks) used in this study. All observers used the same set of definitions established by Venatrack Ltd and based on industry standards (namely OPTA, Prozone) and previous research [5,18]. A copy of the full definition list can be obtained on request.

### Data analysis

Firstly, due to the hierarchical structure of the data, multi-level modelling (see following sentences for further details) was use to predict the activity profiles across different score lines with each of the match-related and performance-related variables (MLwiN v 2.22, Bristol University, Bristol, UK). In this hierarchical structure two levels were used. The top level (level 2) was taken as the variation associated with each game (that comprised of 380 games in this particular season) with level 1 taken as goal difference (e.g. -2, -1, 0, +1, +2 etc.) throughout the entire 90 minutes. The benefit of this hierarchical structure means that, unlike traditional longitudinal data analysis techniques such as repeated-measures ANOVA, the same number of measurement points per individual are not required. Therefore, due to the variation that occurs between matches in the current data set, this statistical technique is well suited to the current data structure. A multi-level model of this nature is also able to describe the underlying trends of a particular component in the population (the fixed part of the model), as well as modelling the unexplained variation around the mean trend for that component due to individual differences (the random part of the model) [39].

The first stage in this multi-level modelling analysis approach was to create a model that explained changes in the different performance variables selected. These were, passing accuracy, free kick accuracy, corner accuracy and cross accuracy. Each performance variable was modelled in turn. Relevant situational parameters were systematically added to the null model and were accepted or rejected on the basis of, firstly, changes in the model fit; as indicated by a difference in log likelihood between models, and the effect of the variable on the performance variable of players, indicated by z-scores. To investigate the variance between players, the intercept was allowed to vary randomly between players. The effect of score line, defined by goal difference (centred at 0 goals) on each of the four performance variables of players was modelled. Goal difference was entered into the model as a quadratic to allow the performance variables to rise and decline under various goal-difference states. Subsequently, the effect of playing position, the zone on the pitch the activity took place (where applicable e.g. corner only took place in the attacking third); the time scored; the opposition’s ability and the team’s ability were included in the model. Following each analysis, the assumption that variations in intercepts were normally distributed with an average of zero was also assessed visually using normality probability plots [39]. Statistical significance was accepted at the 95% confidence level (*P* < 0.05). Mean ± SD were used to describe the average and variability of the activity profile data.

## Results

A total of 570 players across 376 games were analysed, with the maximum number of appearances from one player being 38 and the minimum being 1 game. Table 1 presents the technical performance for each of the teams included in the analysis across the three match statuses (winning, drawing, losing). The average passing accuracy per player per games was 73.6 ± 5.5% per game. With regards to corners, crosses and free kicks players performed on average 19.7 ± 2.6%, 45.4 ± 8.3% and 63.9 ± 12.1% accuracy respectively.

**Table 1:** Mean percentage accuracy per player for corners, crosses, free kicks and passes for each club included in the analysis in a winning, drawing and losing score line state.

Team	Number Games Played	Number of Players Included	WINNING				DRAWING				LOSING				ALL			
			Corner	Cross	Free kick	Pass	Corner	Cross	Free kick	Pass	Corner	Cross	Free kick	Pass	Corner	Cross	Free kick	Pass
1	38	32	17.4	35.6	76.7	79.9	19.6	69.7	80.0	83.1	28.1	38.6	88.9	81.9	20.4	49.6	79.8	81.9
2	38	27	20.0	51.2	44.2	60.9	20.9	40.3	62.9	67.3	11.9	59.5	64.9	67.4	17.5	50.2	61.0	65.8
3	38	31	20.6	56.7	29.3	62.1	18.9	52.6	32.9	65.6	18.9	42.1	38.2	69.4	19.1	48.1	34.7	66.7
4	38	30	18.3	57.6	52.2	68.9	17.1	24.6	49.1	67.4	19.3	39.2	48.2	74.3	18.4	36.9	49.2	71.1
5	35	29	26.3	46.7	97.8	78.9	14.5	45.7	71.5	78.3	20.4	43.3	75.6	75.0	18.6	45.6	74.1	77.9
6	38	30	20.2	60.0	66.4	71.9	21.9	25.0	62.2	72.9	28.7	32.5	81.1	70.1	23.0	33.8	63.0	71.8
7	37	29	25.5	53.1	76.3	76.7	21.5	34.6	83.4	77.5	15.6	44.7	81.1	79.8	20.4	43.1	79.6	78.1
8	37	25	26.3	50.0	59.6	74.8	19.3	41.8	73.1	76.8	16.2	48.4	65.8	78.5	19.8	45.9	67.6	76.2
9	38	26	19.3	55.6	75.2	78.9	15.7	46.7	75.3	78.9	10.0	40.0	42.9	82.7	16.6	49.2	73.2	79.3
10	38	32	23.4	60.6	79.4	82.3	24.7	55.9	79.3	83.3	15.6	58.3	85.7	77.3	23.8	63.7	77.9	82.6
11	37	27	18.5	37.3	54.8	68.3	24.1	41.7	56.4	69.7	28.1	48.2	62.6	74.3	23.8	41.9	57.5	70.4
12	38	28	19.8	64.1	62.1	70.3	23.9	68.9	46.8	67.0	21.0	35.7	62.5	71.1	21.5	52.4	57.9	69.8
13	38	36	16.1	42.7	38.5	69.0	22.9	32.6	44.4	68.0	15.9	23.8	53.9	71.9	18.1	29.4	47.8	70.2
14	38	23	25.0	40.0	58.1	58.8	18.6	43.8	59.0	59.7	19.5	40.0	58.6	67.5	20.2	41.6	58.7	62.8
15	38	28	16.7	28.8	56.7	63.1	16.9	40.3	54.3	61.1	16.4	22.2	50.0	72.2	16.7	32.9	54.1	68.1
16	38	25	33.8	79.8	59.7	72.1	19.0	52.2	81.0	77.6	20.8	51.9	67.6	79.2	22.5	55.9	72.5	76.4
38	38	31	19.1	56.6	72.8	80.9	13.7	43.1	80.6	78.3	11.7	52.1	69.6	79.1	14.8	50.8	75.0	79.7
37	37	25	24.3	71.1	64.9	73.1	21.9	32.7	59.6	72.2	15.1	37.9	62.1	77.1	20.1	46.9	62.0	74.6
38	38	24	20.2	60.0	64.6	73.7	17.7	49.9	73.7	72.6	13.1	47.5	77.6	75.6	16.3	50.9	73.7	74.2
37	37	32	20.0	58.3	38.5	67.2	33.3	40.5	65.7	73.7	16.1	32.3	56.2	77.1	21.7	38.4	57.7	74.9
**Mean**	**376**	**570**	**21.5**	**53.3**	**61.4**	**71.4**	**20.3**	**44.1**	**64.6**	**72.6**	**18.1**	**41.9**	**64.7**	**75.1**	**19.7**	**45.4**	**63.9**	**73.6**
SD	0.8	3.3	4.3	12.3	16.2	7.1	4.4	12.0	14.3	6.7	5.4	10.0	14.1	4.6	2.6	8.3	12.1	5.5

Tables 2, 3 and 4 present the final multi-level models for the development of the match-performance characteristics of passing accuracy, free kick accuracy and corner accuracy, for players of different playing positions, in different pitch zones, across different abilities and against different standards of opposition of players in the 376 English Premier League games analysed. The random part of the multi-level models predicted that the fit of all models was improved when the intercept was allowed to vary randomly (*P* < 0.05), as indicated by the between game standard error displayed in Tables 2, 3 and 4.

**Table 2:** Estimated models for passing accuracy recorded as a percentage.

Passing Accuracy Home			Passing Accuracy Away		
Fixed Effects	Coefficient	SE	Fixed Effects	Coefficient	SE
Constant	0.812	0.010	Constant	0.793	0.011
Goal Difference	-0.014	0.002	Goal Difference	-0.012	0.002
Goal Difference^2^	0.005	0.001	Goal Difference^2^	0.003	0.001
Midfielder	0.016	0.005	Midfielder	0.025	0.005
Striker	-0.071	0.006	Striker	-0.064	0.007
Goal Keeper	-0.103	0.012	Goal Keeper	-0.115	0.012
Middle 3^rd^	0.048	0.005	Middle 3^rd^	0.050	0.006
Defending 3^rd^	-0.047	0.006	Defending 3^rd^	-0.067	0.006
Team Ability	-0.007	0.001	Team Ability	-0.006	0.001
Random Effects	Variance	SE	Random Effects	Variance	SE
Between Game (Repeat)	0.083	0.001	Between Game (Repeat)	0.086	0.001
Within Game (Match ID)	0.005	0.000	Within Game (Match ID)	0.005	0.001

*Notes*. Independent intercepts estimates (centered at Goal Difference 0) for each playing position (reference defender), pitch location (reference attacking 3^rd^), team ability (centered at rank 10) and opposition ability (centered at rank 10). For example, calculations using this model please see S1 File.

**Table 3:** Estimated models for corner accuracy recorded as a percentage.

Corner Accuracy Home			Corner Accuracy Away		
Fixed Effects	Coefficient	SE	Fixed Effects	Coefficient	SE
Constant	0.440	0.017	Constant	0.516	0.041
Goal Difference	0.049	0.012	Goal Difference	0.018	0.015
			Goal Difference^2^	0.017	0.006
			Team Ability	-0.008	0.004
Random Effects	Variance	SE	Random Effects	Variance	SE
Between Game (Repeat)	0.186	0.013	Between Game (Repeat)	0.193	0.015
Within Game (Match ID)	0.000	0.008	Within Game (Match ID)	0.003	0.009

*Notes*. Independent intercepts estimates (Centred at Goal Difference 0) for each playing position (reference defender), pitch location (reference attacking 3^rd^), team ability (centred at 10) and opposition ability (centred at 10). For example, calculations using this model please see S1 File.

**Table 4:** Estimated models for free kick accuracy recorded as a percentage.

FreeKick Accuracy Home			FreeKick Accuracy Away		
Fixed Effects	Coefficient	SE	Fixed Effects	Coefficient	SE
Constant	0.508	0.033	Constant	0.555	0.034
Middle 3^rd^	0.378	0.024	Middle 3^rd^	0.345	0.026
Defending 3^rd^	0.482	0.033	Defending 3^rd^	0.408	0.035
Midfielder	0.004	0.023	Midfielder	-0.020	0.024
Striker	-0.106	0.049	Defender	-0.154	0.050
Goal Keeper	-0.311	0.029	Goal Keeper	-0.305	0.030
Team Ability	-0.010	0.002	Team Ability	-0.012	0.002
Random Effects	Variance	SE	Random Effects	Variance	SE
Between Game (Repeat)	0.150	0.005	Between Game (Repeat)	0.159	0.006
Within Game (Match ID)	0.009	0.003	Within Game (Match ID)	0.003	0.002

*Notes*. Independent intercepts estimates (Centred at Goal Difference 0) for each playing position (reference defender), pitch location (reference attacking 3^rd^), team ability (centred at 10) and opposition ability (centred at 10). For example, calculations using this model please see S1 File.

### Passes

Analyses indicated that player passing accuracy both at home and away in relation to goal difference was non-linear and best described with a quadratic term. In general, models predicted that passing accuracy has a “U” shape association with goal difference. Pass accuracy was greatest as teams conceded more goals and the lowest when goal difference was close, specifically when teams increased their goal difference by 1 or 2 goals. When looking at the effect of goal difference on passing accuracy across pitch zone, players passing accuracy was greatest in the middle third, with the defending third reporting the lowest accuracy. Across all three-pitch zones, passing accuracy was highest when teams were losing by the largest margin, with the lowest accuracy highlighted when teams were winning by 1 to 3 goals. This pattern was also predicted for playing position with regards to goal difference. Across positions, strikers reported the lowest passing accuracy, with midfielders showing slightly better accuracy than defenders across all goal differences. As expected passing accuracy was lowest for the lowest ranked teams across all GD’s.

The estimated parameters of passing accuracy that included goal difference as an independent factor can also be seen in Table 2. The table shows that for both at home and away from home, passing accuracy, goal difference, goal difference^2^, playing position, pitch zone and team ability significantly improved the model fit. It is possible to calculate the performance of players of different playing positions; in different pitch locations in different goal differences, for different ranked teams, either playing at home or away using the coefficients from Table 2. Example calculations can be seen in S1 File.

**Fig 1:**
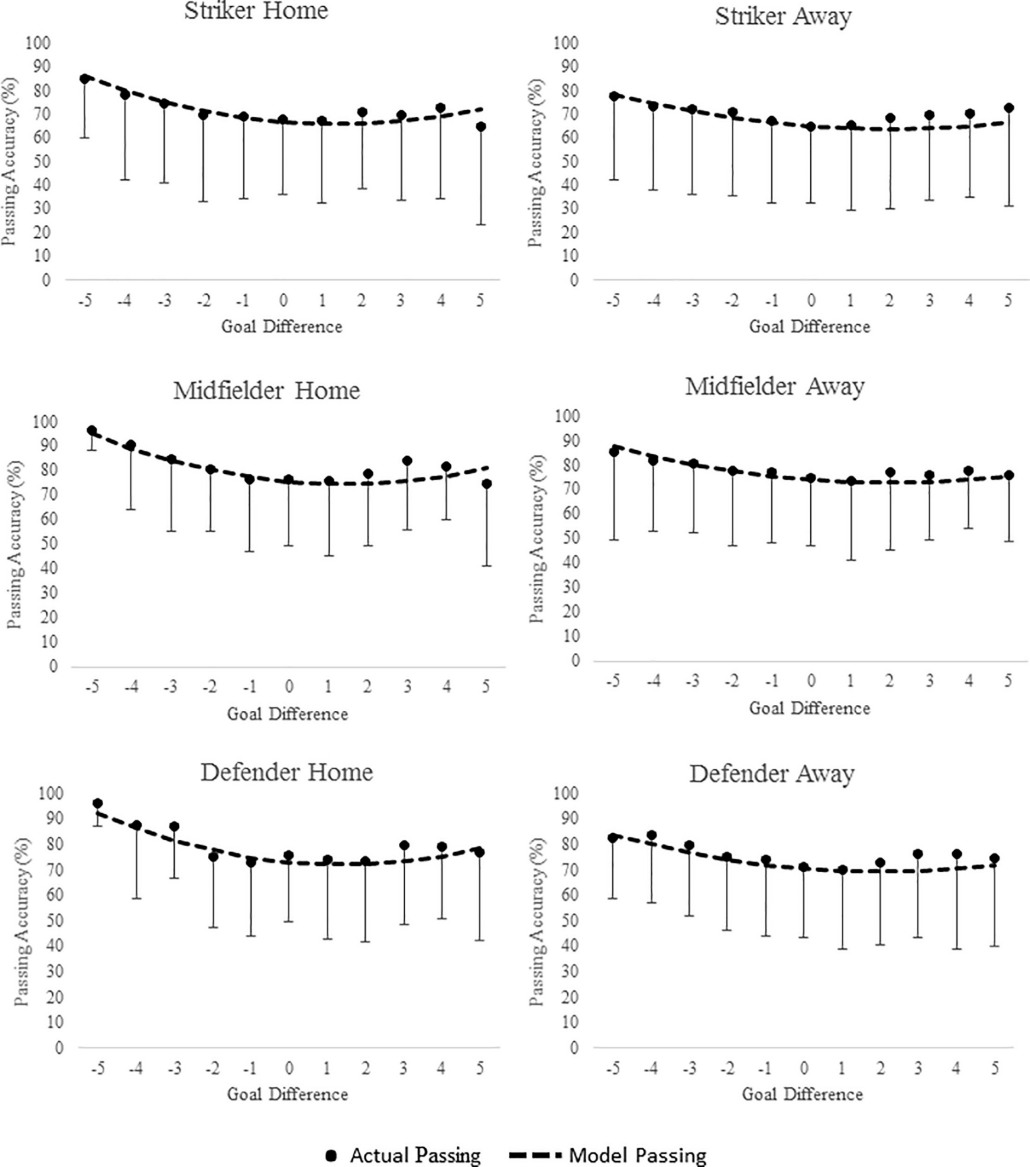
Passing Accuracy (%) during match-play in English Premier League across difference goal differences. Curves are based on predicted passing accuracies from multi-level models of longitudinal data. Points are based on the ‘raw’ passing accuracy data (mean ± SD). Data are presented by playing position both at home and away during match-play.

**Fig 2:**
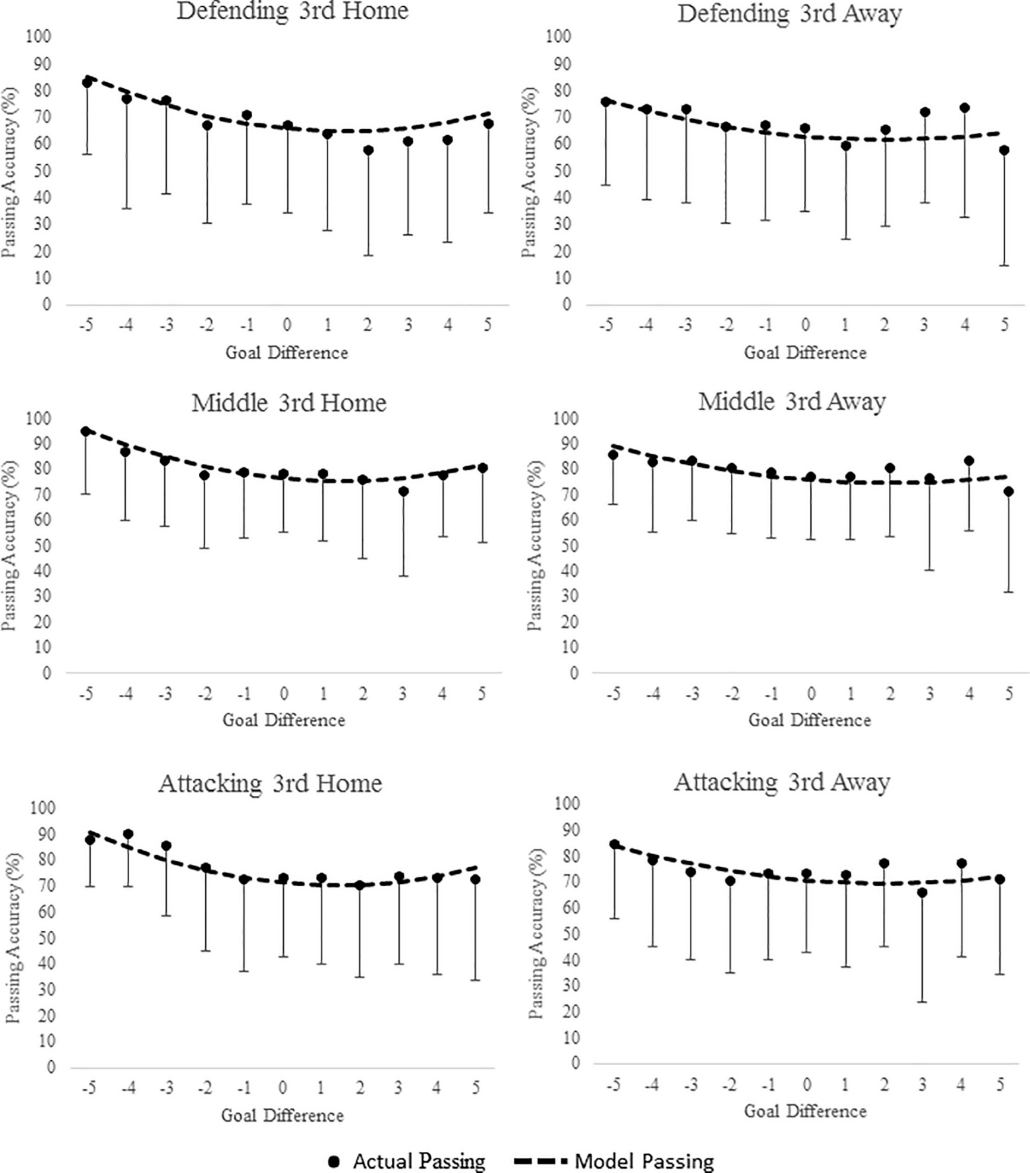
Passing Accuracy (%) during match-play in English Premier League across difference goal differences. Curves are based on predicted passing accuracies from multi-level models of longitudinal data. Points are based on the ‘raw’ passing accuracy data (mean ± SD). Data are presented by pitch location both at home and away during match-play.

**Fig 3:**
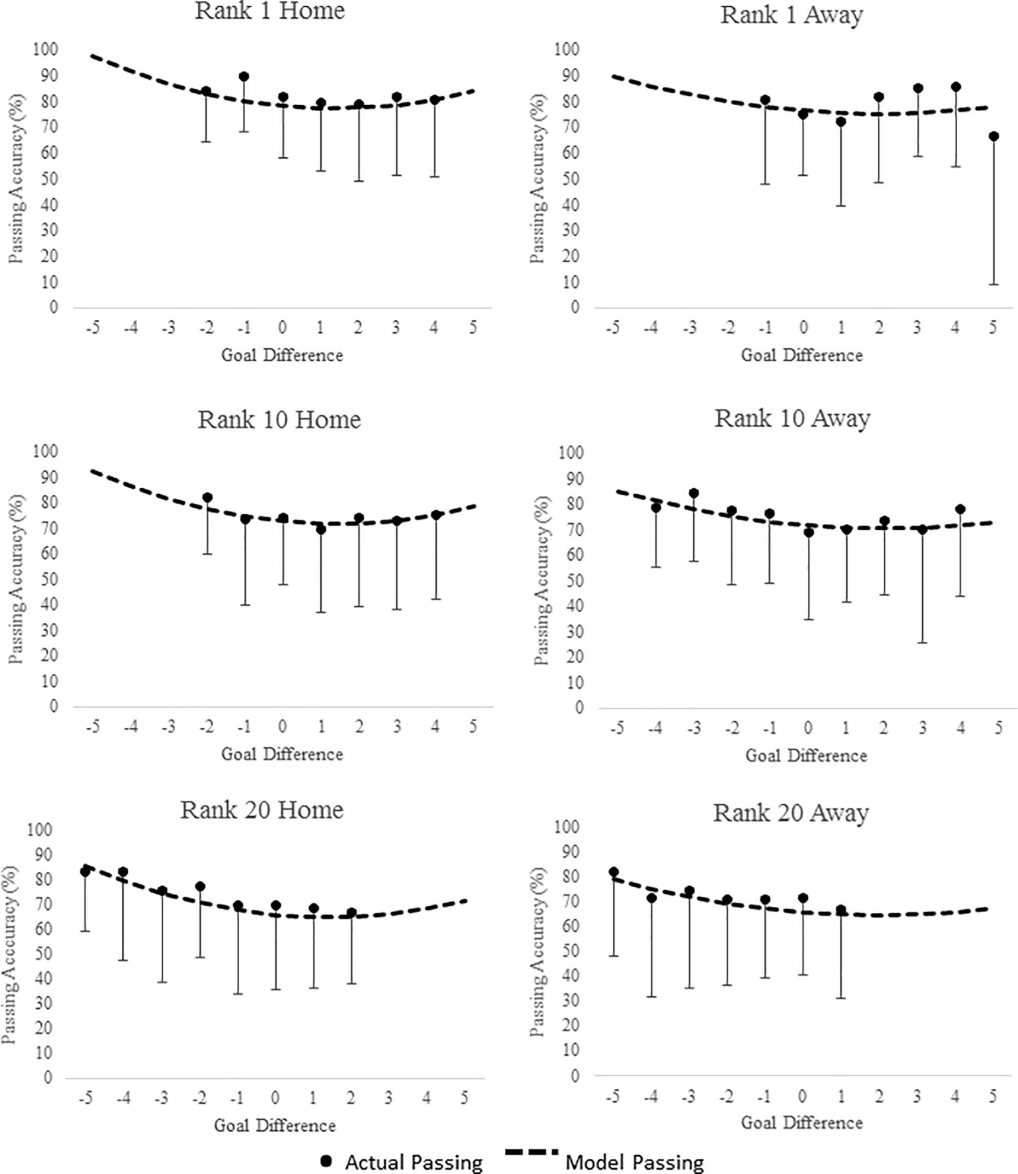
Passing Accuracy (%) during match-play in English Premier League across difference goal differences. Curves are based on predicted passing accuracies from multi-level models of longitudinal data. Points are based on the ‘raw’ passing accuracy data (mean ± SD). Data are presented by Team Rank (final league position) both at home and away during match-play.

S1, S2 and S3 Tables display the mean ± SD of passing accuracy for player position, pitch location and team ability in relation to goal difference. Figs 1, 2 and 3 display the predicted goal-difference related changes in passing accuracy for each playing position, pitch zone and team rank respectively for matches played both at home and away.

### Crosses

Analyses indicated that unlike passing, cross accuracy was not predicted by any of the variables entered into the model (playing position, pitch location, opposition ability, team ability time scored) in relation to goal difference. Cross accuracy was not associated with changes in GD.

### Corners

Analyses indicated that corner accuracy, in relation to goal differences was non-linear and best described with a quadratic term when playing away from home and linear when playing at home. Models predicted that when playing away from home corner accuracy was lowest when the goal difference was close, increasing as teams either scored or conceded goals. Winning teams were found to increase their corner accuracy more rapidly than conceding teams, away from home. When playing at home teams were found to increase their passing accuracy in a linear fashion with the lowest passing accuracy occurring when teams were losing by 5 goals and the greatest when teams were winning by 5 goals.

The estimated parameters for corner accuracy that included goal difference as an independent factor can also be seen in Table 3. The table shows that for corner accuracy away from home, goal difference, goal difference^2^, team ability significantly improved the model fit. For corner accuracy at home only goal difference was found to significantly improve the model fit. It is possible to calculate the performance of players of different ranked teams playing either at home or away using the coefficients from Table 3. S2 Table displays the mean ± SD of corner accuracy for teams of different abilities in relation to goal difference. Example calculations can be seen in S1 File.

### Free kicks

Modelling indicated that free kick accuracy was not predicted by goal difference, however a number of other variables were found to significantly improve the model, when playing at both home and away. In both match environments models predicted that teams free kick accuracy was greatest in the defending 3^rd^ and the lowest in attacking 3^rd^ midfielders were found to have the free kick accuracy with strikers the least. Higher ranked teams were also found to have the highest free kick accuracy. The estimated model for free kick accuracy at both home and away from home can be seen in Table 4. The table shows that for free kick accuracy; team ability, pitch zone and playing position significantly improved the model fit. Example calculations can be seen in S1 File.

**Fig 4:**
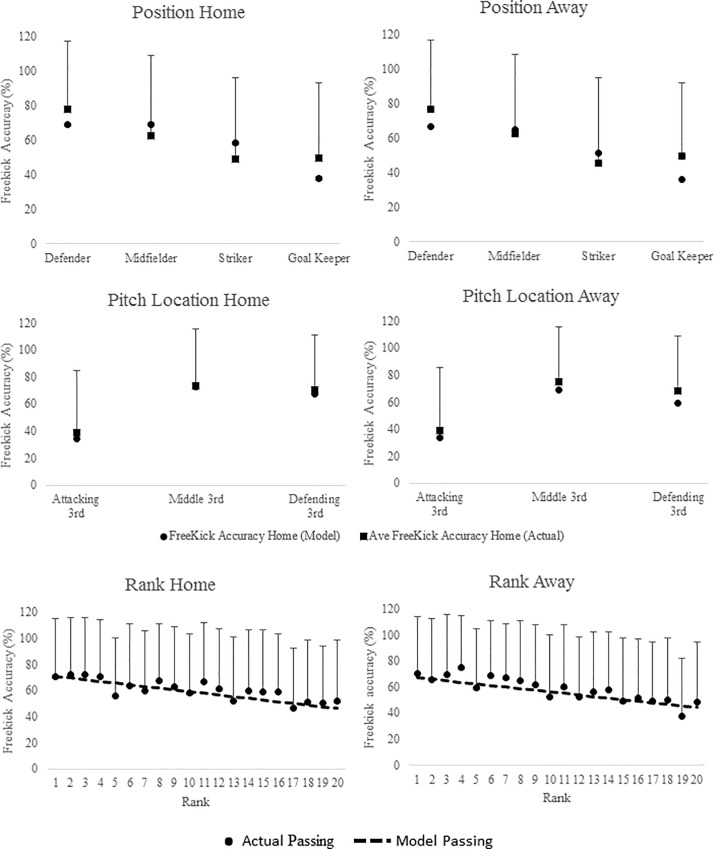
Freekick accuracy (%) during match-play in English Premier League across playing position, pitch location and team ability. Curves are based on predicted passing accuracies from multi-level models of longitudinal data. Points are based on the ‘raw’ freekick accuracy data (mean ± SD). Data are presented by playing position, pitch location and team ability playing both at home and away during match-play.

It is possible to calculate the performance of players of different playing positions, in different pitch locations for different ranked teams, playing either at home or away using the coefficients from Table 4. Fig 4 displays the predicted changes in free kick accuracy for playing position, pitch location and team ability for matches played at both home and away from home.

## Discussion

The aim of the present study was to investigate the effect of playing position, pitch location, team ability and opposition ability on the technical performance of English Premier League players across various goal differences (GD). In support of previous research [2,5,7,9,23], the results suggested that passing accuracy (when playing both at home and away) and corner accuracy (when playing away from home) changed systematically in relation to the goal difference (e.g., winning: +3, +2, +1, drawing: 0, or losing: -1, -2, -3 etc.) in a non-linear manner. Specifically, significant difference between matches, specifically teams showed higher passing accuracies in extremes of GD (e.g., -5 and +5) and the lowest when winning by only a smaller number of goals (e.g., +1 to +3). On the other hand teams were found to have the lowest corner accuracy (away from home) when losing by a small margin (e.g., 1 to 2 goals). Although free kick accuracy was varied across pitch location, playing position and team ability no association was found with goal difference. Crossing accuracy was not found to vary across goal difference or any of the temporal factors considered in the model, suggesting its limited impact on overall team performance and its absence in much of the previous research investigating score line and performance.

### Score line/goal difference

In general, predictive modelling suggested that passing accuracy increased as GD in increased either positively (scoring team) or negatively (conceding team) across all playing positions and pitch locations. At home this increase in passing accuracy was greater when teams conceded goals than when teams scored (e.g., passing accuracy was higher at -4 than +4). Away from home the increase was similar whether teams scored or conceded. The lowest pass accuracy was seen when teams had a small lead specifically a GD of +2 at home and +1 away from home. A number of contrasting results have been highlighted regarding the effect of score line on technical performance variables with studies finding both a winning score line [19,20,40,41,42] and losing score line promoting enhanced play specifically with regards possession and passing success [1,2,8,9,23]. This may explain why at extremes of GD (-3 /+3 and greater), accuracies were similar especially for passing accuracy and corner accuracy away from home (e.g., different teams may react differently to different score lines as shown in the contrasting results of previous studies). For example, Lago-Penas & Dellal [2] and Lago [23] found that as some teams extend their lead they tend to revert to a counterattack or direct style of play, resulting in a reduction in the number of passes in the middle third of the pitch as play is confined to the attacking and defending thirds. Potentially in relation to this, teams have also been found to increase their possession when in a losing state suggesting that they attempt to work harder to get back in the game [2,8,9,30,43]. In contrast, successful teams have been found to maintain possession whether they are winning or losing compared to unsuccessful teams [1]. Alder’s [44] theory suggests that once teams have gained positive momentum through scoring or performing well, they start to ‘cruise’ in an attempt to economise efforts and eventually coast when the goal to be achieved or is within reach, supporting the lowest accuracy seen as teams increased their lead in the current study. Taylor et al. [32] also found that the number of successful passes was higher when losing than when winning, although only one team was analysed for the duration of the 40 matches included. There have also been numerous studies which have reported that a losing match status was associated with greater ball possession (thus suggesting greater passing accuracy) [2,9,23] further supporting the findings of this study.

The term negative facilitation has often been used to describe when teams increase their efforts after failure (e.g. conceding a goal) to overcome negative momentum and get themselves back in the game and could explain the increase in passing seen in the current study as teams concede more goals [45,46]. In contrast to this, positive inhibition is said to explain when teams reduce their effort after periods of success, thus describing the reverse as teams reduce their passing accuracy as they score [45,46]. These contrasting results may explain why for passing accuracy in the current study, both winning and losing by a large margin were found to have the highest passing accuracy, compared to when the GD was close. Using a more sensitive method for measuring score line and including a complete season of games may explain why the current study found that both a winning (e.g., cruising once a substantial lead is achieved) and losing (working hard to get back in the game) score line elicited a higher passing accuracy. This also supports why the lowest passing accuracy occurred when teams were leading by only a goal or 2 (e.g., reverting back to a counterattack style of play to protect their lead) [2,4].

Corner accuracy, when playing away from home was also found to increase at extremes of goal difference (e.g., as teams either extended their lead or conceded more goals). The lowest corner accuracy was reported when the GD was close (e.g., +/- 1 goal). Corner accuracy was also found to increase more rapidly as teams scored goals as opposed to conceding goals. Delgado-Bordonau et al. [47] found teams demonstrate higher averages for offensive variables when they are winning; supporting the increase in successful corners as teams increased their lead. Is it plausible however, that higher successful rates are a function of a team’s superior ability, (hence the winning state) rather than an effect of the match status. Especially as research has also found reductions in attacking variables when teams are in a winning score line [2,4]. The nature of definitions used for score line could explain contrasting results, as most studies have considered score line as an overall status rather than by how many goals a team are winning or losing by, as in the current study.

Although the score line was not found to affect; free kick accuracy both at home or away or corner accuracy playing at home, a number of the temporal factors were significant predictors of technical performance in the respective models and therefore will be discussed later.

The effects of score line were similar whether teams were playing at home or away from home, in terms of significant performance predictors, although teams generally performed better at home (e.g., higher passing, corner, cross, free kick accuracy). This is in support of previous research [1,8,9,30,31] who found home teams had greater possession than their opponents across a range of abilities. Tucker et al. [31] and Taylor et al. [32] also suggested that home teams tended to perform a higher number of attacking actions (goal scored, shots on goal, passes, crosses etc.) which is not surprising given research investigating match location effects has suggested that the home team have a number of advantages over the visiting team [31,33]. Home advantage has also been found to produce triggers for positive momentum (e.g. crowd effects) [34,35,36,37] as supporters are typically in a win frame (e.g. focused on achieving success), thus motivate teams to perform [26], further supporting the findings of the current study.

### Playing position

According to the predictive models, playing position influenced passing accuracy both at home and away across all GD’s. Midfielders performed more accurate passes when playing at both home and away from home than either strikers (10.8% less at home and 7.8% less away from home than midfielders) or defenders (1.6% less at home and 2.5% less away from home). This was consistent across all GD’s.

Although research investigating technical performance differences between players is scarce, especially in different score line states Redwood-Brown et al. [18] did find midfielders and defenders made more passes than attacking players in a case study of one English Premier League team. It was thought attackers maybe less accurate as they have less at stake, if a defender makes a bad pass their error could lead to the opponents scoring, whereas attacking players are able to attempt riskier options in their own attacking third [7,18]. Redwood-Brown et al. [18] also found passing accuracy varied within position (e.g., some midfielders performed better when losing whereas others better when winning). Due to the association between score line, passing accuracy and playing position, there is clearly a need to investigate the performance of individual players in relation to technical performance factors such as passing accuracy. This would enable managers and coaches to apply effective strategies when in each match situation as well as picking the most appropriate team for the predicted match outcome, especially as Redwood-Brown et al. [18] suggested, some players perform better when chasing a lead whereas others like to defend a lead.

Similar to Taylor et al. [5] no differences between playing positions were found for corner success rate in the current study. Evangelos et al. [19] found winners and losers of short-range results (less than one goal difference) had higher rates of corners than wide range results (3 goal difference or more). Thus suggesting teams, who are losing by a small margin, may still adopt an attacking strategy to search for an equaliser. The lack of difference between playing position and cross accuracy were also in contrast to Evangelos et al. [19]. They found midfielders decreased the number of crosses made as they increased their positive goal difference but did not decrease the number of crosses made as they conceded goals (e.g. from a level score no decrease in cross count was seen as teams conceded).

With regards to free kicks midfielders performed more successful free kicks when playing at both home than either strikers (10.9% than midfielders) or defenders (0.4% less than midfielders). Away from home it was defenders who performed the most free kicks closely followed by midfielders (2.2% less than defenders) with strikers performing the least (15.4% less than defenders). As expected goal keepers, due to the nature of the free kicks taken, recorded the lowest free kick accuracy both at home and away compared to outfield players. The only research [5] to examine free kicks in relation to score line, also found differences between playing positions but no score line effect. The incidence of set plays (corners, free kicks, throw ins etc.) maybe more relevant at certain stages of the game rather than in specific goal differences and thus, given the absence of a relationship between set plays and score line suggests they should be investigated in relation to other variables such as tackles, interceptions and regains.

### Pitch zone

Both passing accuracy and free kick accuracy were found to vary across pitch location although free kicks were not affected by goal difference. Teams recorded the highest passing accuracy both at home and away from home in the middle third ahead of both the defending third (16.3% less at home and 9.7% less away from home) and attacking 3^rd^ (9.3% less at home and 3.1% less away from home). Although research investigating the interaction of score line and pitch locations is scarce, Lago [23] found teams passing accuracy was more accurate in the middle third with the worst passing accuracy occurring in the defending third similar to the current study. Lago [23] also found teams changed the amount of time spent in each pitch zone depending on the score line, e.g., when teams were behind they spent more time in the attacking third than when in the lead suggesting teams alter their tactics depending on the evolving score. Research has also suggested [9,48] that successful teams are less likely to deviate from their strategy than unsuccessful teams regardless of the score line and thus possession across pitch location does not change. James et al. [49] teams react to the opposition strategy rather than dictating their own, although the absence of opposition ability in any of the predicting models in the current study suggests performance is more closely related to other factors (e.g. team ability, GD, position etc.)

The current study found that teams higher in ability performed better across a number of the variables investigated, however due to strategic changes, it is not always possible to link those directly to score line changes. Future research would benefit from linking observed strategy (e.g. formation/style of play) with score line and thus performance changes in order to establish how teams respond and thus perform across different GD’s.

In contrast to passing accuracy Freekicks were most accurate in the defending third than either the attacking (48.1% less at home and 40.8% less away from home) and middle third (10.3% less at home and 6.3% less away from home). Possibly, when in the defending third, there is more at stake if the free kick is not cleared by the defending team. In the defending third the opposition is most likely to be down field in anticipation of the ball being cleared as opposed to the attacking third where attacking teams are likely to be setting up for a shot on target. Although pitch location was added to the predictive models of both cross accuracy and corner accuracy it was not found to have a significant effect on these technical performance variables and was thus not included in the final models.

### Team ability

Team ability was found to predict passing and freekick accuracy at home and away as well as corner accuracy away from home. With all three technical performance variables, as expected, higher ranked teams were more accurate than lower ranked teams. This equated to 0.9% less accurate per rank for passing at home, 0.6% less accurate per rank for passing away from home, 1.0% less accurate per rank for freekicks at home, 0.8% less accurate per rank for free kicks away from home and 1.2% less accurate per rank for corners away from home. Although studies [5,8,9,48] have found higher ranked teams perform significantly better than lower ranked teams this has generally been limited to passing accuracy and/or possession strategies. These studies which have generally found successful teams have longer possessions [1,23] and perform more successful passes (due to increase in possession) than unsuccessful teams [8,19,20,24,42]. Hughes and Franks [48] and Harrop and Nevill [50] found teams of lower ability find it harder to achieve success using a possession style of play, potentially due their inability to keep hold of the ball, and thus adopt more of a direct style of play which is indicative of a lower passing accuracy. Lago-Penas and Dellal [2] found that higher ranked teams had less variation in performance than lower ranked teams suggesting that higher ranked teams are able to maintain their performance regardless of the environment and situation (playing at home or away/losing, winning, drawing). They also suggested that teams tended to employ different tactics depending on the characteristics of the players, team formation and philosophy of the team. This may explain why teams in the current study decreased their passing accuracy once they had a convincing lead, adopting a counterattack style to protect their lead. Losing teams on the other hand increase their passing accuracy as GD increased, suggesting they maintain possession and attacking play in search for goals [8].

In support of previous research [5], set plays (corners, free kicks) did not vary as a function of the situation and maintained a stable accuracy across all GD’s. Perhaps highlighting that teams higher in ability are more likely to maintain their strategy regardless of the evolving score line.

### Opposition ability

Opposition ability was not found to influence any of the technical performance variables across different score line states. This is not surprising given that the majority of studies investigating successful and successful teams have found that successful teams show greater passing accuracy regardless of the level of opposition played [1,19,20,23,24,42].

Taylor et al. [5] also found no effect of quality of opposition on the technical aspects of performance (aerial challenges, clearances, passes, crosses, dribbles interceptions, tackles, freekicks, throw ins, corners, shots on target). However, it was suggested that their strong-weak dichotomy was not sensitive enough to show changes in behaviour of the one team used. The sensitive opposition definition used in the current study continue to further cement that opposition ability does not affect technical performance variables regardless of score line. The findings suggest that teams may alter their game strategy (e.g. number of passes, crosses made etc.) in relation to the standard of opposition played but not necessarily their performance accuracy of these technical variables. Future research should therefore consider both frequencies and accuracies of technical performance variables in relation to score line in order to establish how the opposition strategy may effect team’s performance. Considering the psychological impact of important game events (such as goals) is also important, as weaker opponents generally perceive events to have a bigger impact on performance than stronger teams [51]. This is especially important for managers and coaches as negative events (such as conceding goals) have been reported to have a much greater impact on performance than positive events of the same value [25,52,53] and thus could influence team performance.

## Limitations

Although the definition used for score line in the current study was more sensitive than the traditional win, loss, draw it did not give an indication to the actual evolving score line; e.g. 2–0 could be perceived by players differently to 4–2 but would have the same GD. This should therefore be investigated in future research. Another consideration/limitation of the current study was the number of pitch zones used. Although pitch location was included in the multi-level modelling, unlike more recent studies only 3 zones were used. Splitting the pitch further (e.g., nine or twelve zones) would further highlight any variation between pitch zone. Adding additional playing positions (e.g., into wide and central midfielder) would also help to highlight differences between playing positions. It would also be interesting to investigate the extent that individual differences contribute to the overall team, or in this case, the overall mean of their playing position given research [54,55] has suggested variability between players with regards performance accomplishments and success and failure.

## Conclusion

Although previous studies have investigated the effect of score line on player performance, few have considered score line outside of match status (e.g. winning, drawing, and losing). The current study was the first to consider a more sensitive score line with a large data set (35’000 rows of data) including an entire season of data from every team in the English Premier League. The current study also considered a much greater number of matches across one season in an attempt to eradicate the high match-to-match variation. By using only one validated system and an entire season of games more generalisations can also be made by reducing the error seen when trying to compare multiple measurement systems [23].

In support of the majority of previous research [1,3,8,9,25,31,43], which has only examined situational factors independently, the current study extends our understanding of the complex dynamic nature of soccer in relation to technical performance. More research using GD/score line as a foundation is needed to understand the interaction of technical performance variation in different score line states [9]. There is also a great deal of contrasting research with regards to how teams perform when in different score line states, which suggests players may not play the same across all matches [5,9,23,31,49]. Approaching soccer performance as a physical, technical or tactical concept maybe a thing of the past and considering the psychological impact of situational factors and score line may provide more information for managers and coaches to use in order to maximise team performance.

## Supporting information

S1 File Prediction equation examples. (PDF)

S1 Table Mean and SD for all technical performance variables of different playing positions. (PDF)

S2 Table Mean and SD for all technical performance variables of different pitch locations. (PDF)

S3 Table Mean and SD for all technical performance variables of different ranked teams. (PDF)
