# Supplementary material for: Effects of playing position, pitch location, opposition ability and team ability on the technical performance of elite soccer players in different score line states
Source: PLoS One. 2019 Feb 5;14(2):e0211707. doi: 10.1371/journal.pone.0211707 (PMC6363179; doi:10.1371/journal.pone.0211707)
Supplement: S1 File — (PDF) [file pone.0211707.s001.pdf]

S1 File. Prediction Equation Examples

Prediction equations associated with Tables 2, 3 and 4 are given below:

1. Prediction equation for pass accuracy for a midfielder in the middle 3rd, playing for a team ranked 10th, who are losing by 2 goals, away from home is: Pass Accuracy = Constant + (β_1 * goal difference centered at 0) + (β_2 * goal difference centered at 0^2) + (β_3 * midfielder) + (β_4 * middle 3rd) + (β_5 * team ability centered at rank 10) which is: 0.793 + (-0.012*-2) + (0.003*-2^2) + (0.025) + (0.050) + (-0.006*9) = 0.838 resulting in a passing accuracy of 83.8%.

2. The prediction equation for corner accuracy for a team ranked 10th, who are winning by 2 goals away from home is: Corner Accuracy = Constant + (β_1 * goal difference centered at 0) + (β_2 * goal difference centered at 0^2) + (β_3 * team ability centered at rank 10) which is:0.516 + (0.018 * 2) + (0.017 * 2^2) + (-0.008*9) =0.481 resulting in a corner accuracy of 48.1%.

3. The prediction equation for free kick accuracy playing at home, in the middle 3rd, for a striker playing for a team ranked 10th in all goal differences is: Constant + (β_1 * middle 3rd) + (β_2 *striker) + (β_3 * team ability rank 10) which is: 0.508 + (0.378) + (-0.106) + (-0.010*9) = 0.69 resulting in a free kick accuracy of 69.0%.
