# Supplementary material for: Effects of playing position, pitch location, opposition ability and team ability on the technical performance of elite soccer players in different score line states
Source: PLoS One. 2019 Feb 5;14(2):e0211707. doi: 10.1371/journal.pone.0211707 (PMC6363179; doi:10.1371/journal.pone.0211707)
Supplement: S1 Table — (PDF) [file pone.0211707.s002.pdf]

S1 Table. Mean and SD for all technical performance variables of different playing positions.

Goal Difference		Passing Accuracy (%)		Cross Accuracy (%)		Corner Accuracy (%)		FreeKick Accuracy (%)	
-5	Striker	84.9 ± 24.9	77.5 ± 35.3	0.0 ± 0.0	100 ± 0.0		100.0 ± 0.0		
	Midfielder	90.0 ± 13.6	75.4 ± 36.3	30.6 ± 40.0	40.0 ± 54.8	50.0 ± 70.7	50.0 ± 70.7	75.0 ± 35.3	33.3 ± 57.8
	Defender	87.1 ± 24.4	82.6 ± 24.0	0.0 ± 0.0	13.3 ± 29.8			100.0 ± 0.0	77.8 ± 44.1
-4	Striker	80.7 ± 31.3	73.5 ± 35.6	0.0 ± 0.0	14.3 ± 37.8			100.0 ± 0.0	100.0 ± 0.0
	Midfielder	87.8 ± 26.5	81.7 ± 29.0	0.0 ± 0.0	13.6 ± 32.3	0.0 ± 0.0	20.0 ± 44.7	58.3 ± 49.2	62.5 ± 43.3
	Defender	84.5 ± 28.9	83.8 ± 27.0	12.5 ± 35.4	15.0 ± 33.7			100.0 ± 0.0	72.2 ± 44.1
-3	Striker	72.1 ± 34.9	72.1 ± 35.8	28.6 ± 48.8	0.0 ± 0.0	100.0 ± 0.0	100.0 ± 0.0	100.0 ± 0.0	25.0 ± 50.0
	Midfielder	82.7 ± 29.4	80.5 ± 28.5	34.6 ± 46.5	9.3 ± 23.6	30.9 ± 46.1	46.7 ± 47.9	65.0 ± 47.4	59.2 ± 49.1
	Defender	82.5 ± 26.9	79.7 ± 27.8	15.4 ± 37.6	10.0 ± 30.5		50.0 ± 70.7	75.0 ± 46.2	78.6 ± 40.7
-2	Striker	69.8 ± 35.8	70.9 ± 35.3	6.7 ± 23.2	22.5 ± 39.8	0.0 ± 0.0.	65.4 ± 42.2	66.7 ± 57.8	81.8 ± 40.4
	Midfielder	77.3 ± 29.2	77.4 ± 30.2	18.4 ± 32.9	13.0 ± 30.5	45.2 ± 45.7	49.6 ± 47.9	40.9 ± 46.9	55.0 ± 47.5
	Defender	78.5 ± 27.8	75.3 ± 29.1	15.3 ± 34.2	20.4 ± 38.1	66.7 ± 57.7	66.7 ± 44.4	79.8 ± 39.9	83.2 ± 35.1
-1	Striker	69.7 ± 34.1	67.0 ± 34.6	12.2 ± 30.7	16.9 ± 35.7	31.1 ± 42.2	19.7 ± 34.3	46.4 ± 49.9	25.0 ± 42.9
	Midfielder	76.2 ± 29.6	77.2 ± 29.3	17.7 ± 32.9	21.1 ± 37.3	33.1 ± 40.9	45.3 ± 46.4	60.5 ± 47.7	58.1 ± 48.0
	Defender	74.7 ± 29.4	74.3 ± 30.4	20.6 ± 35.7	22.6 ± 39.2	35.9 ± 46.2	25.0 ± 46.2	76.7 ± 41.6	81.6 ± 37.5
0	Striker	67.2 ± 31.3	64.6 ± 32.5	17.2 ± 34.2	18.7 ± 36.4	43.3 ± 43.2	50.4 ± 44.0	43.8 ± 47.6	40.4 ± 48.5
	Midfielder	75.4 ± 27.2	74.8 ± 27.8	19.6 ± 34.7	21.3 ± 38.1	46.0 ± 38.9	44.4 ± 43.7	60.4 ± 46.6	61.6 ± 45.5
	Defender	74.9 ± 26.5	71.2 ± 27.9	22.4 ± 37.6	17.0 ± 32.7	28.9 ± 40.9	40.2 ± 48.3	80.1 ± 38.3	74.0 ± 40.9
1	Striker	68.4 ± 34.3	65.1 ± 35.7	29.6 ± 44.0	14.1 ± 33.1	61.1 ± 50.2	67.9 ± 46.4	59.5 ± 49.0	50.0 ± 51.6
	Midfielder	76.1 ± 30.7	73.2 ± 32.0	21.9 ± 37.1	23.7 ± 38.9	52.9 ± 45.6	48.2 ± 45.5	64.5 ± 46.7	65.9 ± 45.4
	Defender	72.9 ± 31.2	70.0 ± 31.1	25.8 ± 40.3	20.7 ± 37.8	60.0 ± 51.6	50.0 ± 50.0	72.3 ± 42.9	72.8 ± 43.3
2	Striker	71.1 ± 33.1	68.1 ± 37.9	14.2 ± 33.7	35.0 ± 46.1	80.3 ± 35.6	57.1 ± 53.5	33.3 ± 51.6	50.0 ± 54.8
	Midfielder	78.7 ± 29.5	76.9 ± 32.0	26.9 ± 41.8	16.4 ± 35.7	62.6 ± 44.1	65.5 ± 41.4	72.6 ± 41.5	82.0 ± 37.2
	Defender	74.8 ± 31.8	72.7 ± 32.4	23.1 ± 39.9	18.5 ± 37.1	62.5 ± 47.9	12.5 ± 17.7	77.6 ± 40.4	76.6 ± 41.5
3	Striker	73.7 ± 32.7	69.6 ± 36.2	14.3 ± 36.3	0.0 ± 0.0	76.7 ± 25.1		50.0 ± 57.7	100.0 ± 0.0
	Midfielder	82.7 ± 28.2	85.8 ± 26.4	14.9 ± 35.2	15.6 ± 35.2	44.0 ± 47.8	81.2 ± 37.2	74.1 ± 44.7	100.0 ± 0.0
	Defender	80.4 ± 29.7	76.3 ± 33.2	23.7 ± 38.8	0.0 ± 0.0			90.9 ± 29.4	84.6 ± 37.6
4	Striker	69.8 ± 40.7	70.5 ± 35.4	20.0 ± 44.7	25.0 ± 50.0	0.0 ± 0.0	100.0 ± 0.0	33.3 ± 57.7	100.0 ± 0.0
	Midfielder	88.1 ± 21.9	87.5 ± 23.4	36.4 ± 45.2	50.0 ± 57.7	31.3 ± 45.8	50.0 ± 70.7	87.5 ± 34.2	100.0 ± 0.0
	Defender	82.2 ± 28.3	76.1 ± 37.1	16.7 ± 40.8	33.3 ± 57.7			75.0 ± 45.2	75.0 ± 41.8
5	Striker	74.3 ± 37.6	72.5 ± 41.4	0.0 ± 0.0	0.0 ± 0.0	0.0 ± 0.0	0.0 ± 0.0	0.0 ± 0.0	
	Midfielder	83.3 ± 25.4	85.6 ± 26.7	16.7 ± 40.8	0.0 ± 0.0	66.7 ± 51.6	100.0 ± 0.0	75.0 ± 35.4	100.0 ± 0.0
	Defender	83.0 ± 28.6	74.4 ± 34.5	25.0 ± 35.4				80.0 ± 44.7	
