# Supplementary material for: Effects of playing position, pitch location, opposition ability and team ability on the technical performance of elite soccer players in different score line states
Source: PLoS One. 2019 Feb 5;14(2):e0211707. doi: 10.1371/journal.pone.0211707 (PMC6363179; doi:10.1371/journal.pone.0211707)
Supplement: S2 Table — (PDF) [file pone.0211707.s003.pdf]

S2 Table. Mean and SD for all technical performance variables of different pitch locations.

		HOME	Away	Home	Away	Home	Away	Home	Away
Goal Difference		Passing Accuracy (%)		Cross Accuracy (%)		Corner Accuracy (%)		FreeKick Accuracy (%)	
-5	Attacking 3rd	87.1 ± 21.7	87.4 ± 32.5	26.2 ± 38.3	48.9 ± 38.9			50.0 ± 0.0	0.0 ± 0.0
	Middle 3rd	91.5 ± 15.5	89.5 ± 28.6	0.0 ± 0.0					89.5 ± 28.6
	Defending 3rd	82.8 ± 26.6	78.4 ± 29.8						75.0 ± 50.0
-4	Attacking 3rd	86.9 ± 26.3	81.3 ± 31.2	0.0 ± 0.0	18.2 ± 36.3			75.0 ± 35.4	64.3 ± 47.6
	Middle 3rd	89.2 ± 20.1	86.3 ± 22.7		0.0 ± 0.0			71.4 ± 48.8	67.9 ± 42.1
	Defending 3rd	74.0 ± 39.2	75.2 ± 33.0					100.0 ± 0.0	64.3 ± 47.6
-3	Attacking 3rd	86.6 ± 22.5	76.7 ± 31.5	26.0 ± 43.1	8.7 ± 24.8			56.3 ± 49.6	35.2 ± 47.7
	Middle 3rd	85.9 ± 20.7	82.5 ± 24.2	40.0 ± 54.8	14.3 ± 36.3			81.8 ± 40.5	79.8 ± 39.9
	Defending 3rd	75.4 ± 35.2	72.9 ± 34.9					50.0 ± 54.8	72.6 ± 40.8
-2	Attacking 3rd	79.2 ± 29.1	71.5 ± 34.2	15.5 ± 31.7	32.9 ± 15.5			38.9 ± 46.5	32.1 ± 44.7
	Middle 3rd	84.4 ± 24.0	81.1 ± 24.4	13.9 ± 33.5	29.6 ± 46.5			60.4 ± 47.9	76.4 ± 39.9
	Defending 3rd	67.6 ± 35.4	67.3 ± 35.7	0.0 ± 0.0	0.0 ± 0.0			64.5 ± 43.9	71.6 ± 41.1
-1	Attacking 3rd	74.8 ± 31.3	74.0 ± 32.5	16.1 ± 31.4	22.3 ± 38.1			29.4 ± 42.9	36.3 ± 46.4
	Middle 3rd	83.0 ± 19.2	79.2 ± 25.3	22.3 ± 41.6	21.1 ± 40.3			75.7 ± 41.5	76.8 ± 40.8
	Defending 3rd	69.7 ± 33.7	67.3 ± 34.3	5.0 ± 15.8	12.0 ± 31.6			73.9 ± 59.7	67.3 ± 42.0
0	Attacking 3rd	72.9 ± 29.1	71.7 ± 31.3	20.2 ± 34.9	18.8 ± 34.9			37.9 ± 44.9	36.2 ± 44.9
	Middle 3rd	80.6 ± 18.0	76.5 ± 23.8	22.3 ± 41.2	26.9 ± 43.9			75.5 ± 41.1	73.9 ± 40.7
	Defending 3rd	67.7 ± 31.9	64.1 ± 30.8	30.2 ± 44.8	8.2 ± 24.5			74.8 ± 38.1	67.1 ± 39.6
1	Attacking 3rd	73.9 ± 32.9	72.5 ± 35.0	23.8 ± 38.6	20.6 ± 36.7			45.8 ± 47.9	44.6 ± 47.8
	Middle 3rd	81.5 ± 23.1	76.3 ± 26.6	30.2 ± 45.8	25.7 ± 44.3			76.8 ± 40.4	70.2 ± 43.8
	Defending 3rd	64.5 ± 35.3	61.7 ± 33.9	0.0 ± 0.0	0.0 ± 0.0			58.7 ± 44.1	54.1 ± 43.7
2	Attacking 3rd	73.9 ± 33.1	77.3 ± 32.3	23.3 ± 39.7	23.4 ± 40.4			51.6 ± 47.9	60.0 ± 50.3
	Middle 3rd	81.2 ± 25.8	80.6 ± 27.5	28.9 ± 45.1	0.0 ± 0.0			80.6 ± 36.9	76.8 ± 40.7
	Defending 3rd	69.3 ± 34.9	64.9 ± 36.5	0.0 ± 0.0	33.3 ± 57.8			63.7 ± 42.3	50.9 ± 46.8
3	Attacking 3rd	76.8 ± 34.5	76.1 ± 35.9	18.6 ± 36.9	8.3 ± 25.7			43.8 ± 51.2	100 ± 0.0
	Middle 3rd	83.7 ± 26.0	83.2 ± 28.3	33.3 ± 57.8	0.0 ± 0.0			90.0 ± 30.3	90.0 ± 30.8
	Defending 3rd	71.4 ± 34.4	66.2 ± 36.8	0.0 ± 0.0				58.3 ± 47.9	54.2 ± 49.8
4	Attacking 3rd	77.7 ± 36.5	76.5 ± 35.0	28.6 ± 43.5	36.4 ± 50.5			42.9 ± 53.5	
	Middle 3rd	84.5 ± 28.0	84.3 ± 25.7					90.0 ± 30.8	95.0 ± 15.8
	Defending 3rd	69.3 ± 38.9	66.6 ± 42.0					57.1 ± 53.4	65.7 ± 57.8
5	Attacking 3rd	83.2 ± 27.3	76.4 ± 32.6	13.6 ± 32.3	14.3 ± 37.8			16.7 ± 28.9	100.0 ± 0.0
	Middle 3rd	87.2 ± 20.5	85.6 ± 27.3	0.0 ± 0.0				66.7 ± 57.8	
	Defending 3rd	70.8 ± 39.3	67.1 ± 40.4					100.0 ± 0.0	
