# Supplementary material for: Effects of playing position, pitch location, opposition ability and team ability on the technical performance of elite soccer players in different score line states
Source: PLoS One. 2019 Feb 5;14(2):e0211707. doi: 10.1371/journal.pone.0211707 (PMC6363179; doi:10.1371/journal.pone.0211707)
Supplement: S3 Table — (PDF) [file pone.0211707.s004.pdf]

S3 Table. Mean and SD for all technical performance variables of different ranked teams.

		HOME	Away	Home	Away	Home	Away	Home	Away
Goal Difference		Passing Accuracy (%)		Cross Accuracy (%)		Corner Accuracy (%)		FreeKick Accuracy (%)	
-5	Rank 1								
	Rank 10								
	Rank 20	83.3 ± 24.4	81.9 ± 33.7	8.3 ± 20.4	0.0 ± 0.0			75.0 ± 35.3	
-4	Rank 1								
	Rank 10		78.8 ± 23.7		0.0 ± 0.0		50.0 ± 70.7		75.0 ± 50.0
	Rank 20	83.2 ± 35.6	71.3 ± 39.8	0.0 ± 0.0	0.0 ± 0.0		50.0 ± 70.7		0.0 ± 0.0
-3	Rank 1								
	Rank 10		84.4 ± 27.0		3.7 ± 11.1		66.7 ± 57.7		66.7 ± 31.6
	Rank 20	75.5 ± 36.9	74.4 ± 39.0	9.1 ± 30.2	20.8 ± 40.1	33.3 ± 51.6		62.5 ± 51.8	50.0 ± 70.7
-2	Rank 1	84.3 ± 20.1		25.0 ± 50.0		33.3 ± 57.7		50.0 ± 70.7	
	Rank 10	81.9 ± 21.9	77.7 ± 29.3	50.0 ± 70.7	16.7 ± 40.8	33.3 ± 57.7	44.4 ± 50.9	66.7 ± 47.1	58.3 ± 49.2
	Rank 20	77.0 ± 28.2	70.6 ± 34.5	17.7 ± 34.8	14.3 ± 37.8	33.3 ± 51.6	50.0 ± 0.0	61.4 ± 45.0	35.7 ± 47.6
-1	Rank 1	89.7 ± 21.7	80.9 ± 33.1	8.1 ± 16.2	0.0 ± 0.0	25.0 ± 31.9	66.7 ± 57.7	60.0 ± 54.8	28.6 ± 48.8
	Rank 10	73.7 ± 33.8	76.2 ± 27.2	30.0 ± 48.3	7.9 ± 25.0	0.0 ± 0.0	41.7 ± 49.2	33.3 ± 43.3	68.8 ± 46.2
	Rank 20	69.5 ± 35.6	71.1 ± 31.8	25.4 ± 42.0	14.4 ± 34.9	0.0 ± 0.0	34.7 ± 45.2	57.4 ± 49.6	56.8 ± 46.6
0	Rank 1	81.8 ± 23.9	75.3 ± 24.0	16.4 ± 31.1	16.7 ± 34.8	46.5 ± 48.6	46.1 ± 45.1	70.9 ± 44.3	76.2 ± 40.6
	Rank 10	74.3 ± 26.3	68.8 ± 34.2	22.2 ± 38.1	20.0 ± 42.2	24.2 ± 31.4	50.0 ± 47.4	57.7 ± 46.5	66.1 ± 43.4
	Rank 20	69.4 ± 33.5	71.7 ± 31.3	35.8 ± 44.8	29.5 ± 43.0	54.5 ± 47.2	25.0 ± 42.5	76.1 ± 38.7	52.8 ± 46.2
1	Rank 1	79.4 ± 26.6	72.1 ± 32.8	20.1 ± 36.3	20.0 ± 36.8	61.1 ± 46.9	33.3 ± 57.7	80.7 ± 39.2	65.9 ± 45.1
	Rank 10	69.6 ± 32.9	69.9 ± 28.3	21.7 ± 36.4	28.1 ± 40.7	50.0 ± 50.0	66.7 ± 47.1	62.6 ± 46.4	66.2 ± 42.3
	Rank 20	68.3 ± 32.1	66.8 ± 35.7	14.3 ± 24.4	28.5 ± 48.8	33.3 ± 57.7	50.0 ± 44.7	53.3 ± 50.6	20.0 ± 44.7
2	Rank 1	78.8 ± 29.8	81.8 ± 33.4	30.0 ± 48.3	100.0 ± 22.3	50.0 ± 57.7	50.0 ± 0.0	70.0 ± 44.7	75.0 ± 50.0
	Rank 10	74.3 ± 34.9	73.7 ± 29.2		33.3 ± 57.7	100.0 ± 0.0	50.0 ± 0.0	87.5 ± 25.0	62.5 ± 47.9
	Rank 20	66.8 ± 28.6		0.0 ± 0.0		66.7 ± 57.7		41.7 ± 49.2	
3	Rank 1	81.8 ± 30.4	85.5 ± 26.7	11.1 ± 33.3	0.0 ± 0.0	100.0 ± 0.0	100.0 ± 0.0	100.0 ± 0.0	100.0 ± 0.0
	Rank 10	72.9 ± 34.6	70.0 ± 44.7	16.7 ± 40.8		0.0 ± 0.0		41.7 ± 52.0	
	Rank 20								
4	Rank 1	80.9 ± **29.9**	85.6 ± 30.9	16.7 ± 28.7	100.0 ± 0.0	0.0 ± 0.0		0.0 ± 0.0	100.0 ± 0.0
	Rank 10	75.1 ± 33.0	77.8 ± 34.2						100.0 ± 0.0
	Rank 20								
5	Rank 1		66.7 ± 57.8						
	Rank 10								
	Rank 20								
